# Coffee consumption is not associated with increased risk of atrial fibrillation: results from two prospective cohorts and a meta-analysis

**DOI:** 10.1186/s12916-015-0447-8

**Published:** 2015-09-23

**Authors:** Susanna C. Larsson, Nikola Drca, Mats Jensen-Urstad, Alicja Wolk

**Affiliations:** Unit of Nutritional Epidemiology, Institute of Environmental Medicine, Karolinska Institutet, Stockholm, Sweden; Department of Cardiology, Karolinska University Hospital, Karolinska Institutet, Stockholm, Sweden

## Abstract

**Background:**

Whether coffee consumption affects the risk of developing atrial fibrillation (AF) remains unclear. We sought to investigate the association between coffee consumption and incidence of AF in two prospective cohorts, and to summarize available evidence using a meta-analysis.

**Methods:**

Our study population comprised 41,881 men in the Cohort of Swedish Men and 34,594 women in the Swedish Mammography Cohort who had provided information on coffee consumption in 1997 and were followed up for 12 years. Incident cases of AF were ascertained by linkage with the Swedish Hospital Discharge Register. For the meta-analysis, prospective studies were identified by searching PubMed and Embase through 22 July 2015, and by reviewing the reference lists of retrieved articles. Study-specific relative risks were combined using a random effects model.

**Results:**

We ascertained 4,311 and 2,730 incident AF cases in men and women, respectively, in the two cohorts. Coffee consumption was not associated with AF incidence in these cohort studies. The lack of association was confirmed in a meta-analysis, including six cohort studies with a total of 10,406 cases of AF diagnosed among 248,910 individuals. The overall relative risk (95 % confidence interval) of AF was 0.96 (0.84–1.08) for the highest versus lowest category of coffee consumption, and 0.99 (0.94–1.03) per 2 cups/day increment of coffee consumption.

**Conclusions:**

We found no evidence that coffee consumption is associated with increased risk of AF.

**Electronic supplementary material:**

The online version of this article (doi:10.1186/s12916-015-0447-8) contains supplementary material, which is available to authorized users.

## Background

Coffee is a complex beverage containing many different chemical substances that may affect health [1]. Moderate coffee consumption has been associated with reduced risk of type 2 diabetes [2], coronary heart disease [3], stroke [3, 4], and mortality from all causes and cardiovascular disease [5]. Whether coffee consumption also influences the risk of atrial fibrillation (AF), the most frequent cardiac arrhythmia, remains unclear. A major compound in coffee is caffeine, which is a vasoactive substance that can promote the release of norepinephrine and epinephrine [6]. Short-term metabolic studies in humans performed in the 1970s and 1980s showed that acute caffeine ingestion increased plasma renin activity, catecholamine concentrations and blood pressure [7], as well as shortened the refractory period of the right atrium, AV node and the right ventricle, and prolonged the refractory period of the left atrium [8]. Several more recent randomized controlled trials have reported that caffeine intake produces an acute increase in blood pressure (mean change of 8 mm/Hg and 6 mm/Hg in systolic and diastolic blood pressure, respectively, within <60–180 min after intake of 200–300 mg caffeine or coffee) [9], whereas long-term (1–4 months) coffee consumption has no effect on blood pressure [10]. Two recent meta-analyses of observational studies of caffeine intake in relation to AF risk did not support an association between a high caffeine intake and increased AF risk; if anything, an inverse association was indicated [11, 12]. In experimental animal models, caffeine administration has been shown to either reduce [13] or increase the propensity for AF [14].

Patients with AF have a substantially increased risk of stroke, heart failure, dementia and all-cause mortality, and are a high economic burden for the society [15]. Given the serious complications of AF and the fact that coffee is one of the most popular beverages around the world, assessing whether coffee consumption is associated with risk of AF has important public health implications. To further clarify the relationship between coffee consumption and AF incidence, we examined this association in the Cohort of Swedish Men (COSM) and the Swedish Mammography Cohort (SMC). Moreover, we conducted a meta-analysis of available studies of coffee consumption and risk of AF.

## Methods

### Study population

The COSM and the SMC are prospective cohorts of men and women, respectively, in central Sweden. The COSM was initiated in the late autumn of 1997 when all men who were born between 1918 and 1952 and resided in Västmanland and Örebro counties received a mailed questionnaire about diet, beverage consumption, lifestyle, and other risk factors for chronic diseases. A total of 48,850 men (49 % of the source population) answered the questionnaire. Simultaneously, an identical questionnaire (except for some sex-specific questions) was completed by 39,227 women (70 % response rate) who were born between 1914 and 1948 and lived in Västmanland and Uppsala counties. Those women had been enrolled in the SMC in 1987–90, but the baseline data did not include cardiovascular risk factors. We therefore used the 1997 questionnaire as the baseline for the present analyses. We excluded men and women with an incorrect/missing personal identification number (*n* = 297 men and *n* = 243 women), those with a diagnosis of AF (*n* = 1,496 men and *n* = 634 women) or cancer other than non-melanoma skin cancer (*n* = 2,592 men and *n* = 1,811 women) before baseline, those who died between the administration of the 1997 questionnaire and start of follow-up (1 January 1998; *n* = 55 men and *n* = 26 women), and those with missing data on coffee consumption (*n* = 2,529 men and *n* = 1,919 women). This left 41,881 men, 45–79 years of age, and 34,594 women, 49–83 years of age for analysis. The Regional Ethical Review Board at Karolinska Institutet in Stockholm, Sweden, approved the study. The completion of the self-administered questionnaire was considered to imply informed consent.

### Assessment of coffee consumption and covariates

The questionnaire completed by participants in the autumn of 1997 inquired about education, smoking, body weight, height, physical activity, history of hypertension and diabetes, family history of myocardial infarction before 60 years of age, diet, and consumption of coffee and other beverages. The questionnaire assessed average consumption of 96 foods and beverages during the previous year. With regard to coffee consumption, participants were asked to report how many cups of coffee they consumed per day or per week. In a validation study (conducted in a subsample of 111 participants of the SMC) of a similar questionnaire, the questionnaire assessment of coffee correlated well with the mean of four 7-day dietary record assessments (Spearman correlation coefficient, r = 0.61) [16].

Cardiac disease was defined as a diagnosis of ischemic heart disease or heart failure in the Swedish National Inpatient Register. We classified participants as having hypertension and diabetes if they reported in the questionnaire that they had any of these diseases or if they had a diagnosis of hypertension or diabetes in the Swedish Hospital Discharge Register or the Swedish National Diabetes Register. Participants were asked to indicate their time spent on walking and bicycling during the last year. They could choose from six predefined categories: almost never; <20 min/d; 20–40 min/d; 40–60 min/d; 1–1.5 h/d; or ≥1.5 h/d. We calculated body mass index as self-reported weight (in kg) divided by the square of self-reported height (in m). Alcohol (ethanol) consumption, in the last year, was calculated by multiplying the frequency of consumption of beer, wine and liquor by the amount consumed at each occasion.

### Ascertainment of cases

Via linkage of study participants (using the personal identification number assigned to each Swedish citizen) with the Swedish Hospital Discharge Register, we obtained information on dates of diagnosis of AF. Diagnoses in the Hospital Discharge Register are coded according to the Swedish International Classification of Disease (ICD) system. AF was defined as atrial fibrillation or atrial flutter (ICD-10 code I48). A previous validation study of AF diagnoses in the Hospital Discharge Register showed that the AF diagnosis is correct in 97 % of the cases [17].

### Statistical methods

Participants were followed up from 1 January 1998 until the date of diagnosis of AF, date of death (information obtained from the Swedish Death Register) or end of follow-up (31 December 2009), whichever came first. We categorized participants into five groups (approximate quintiles) according to their coffee consumption: <2 cups/d (reference); 2 to <3 cups/d; 3 to <4 cups/d; 4 to <5 cups/d; and ≥5 cups/d. We used Cox proportional hazards regression models with age as the time scale to estimate relative risks (RR) with corresponding 95 % confidence intervals (CI). All multivariable models included education (less than high school; high school; university) and known risk factors for AF, including smoking (never; past and <20 pack-years; past and ≥20 pack-years; current and <20 pack-years; current and ≥20 pack-years), history of cardiac disease (yes or no), history of hypertension (yes or no), history of diabetes (yes or no), body mass index (in kg/m^2^, continuous), walking/bicycling (almost never; <20 min/d; 20–40 min/d; 40–60 min/d; >1 h/d), family history of myocardial infarction (yes or no), and consumption of alcohol (nondrinkers; and <1, 1–6, 7–14 and >14 drinks/wk) and tea (cups/d, continuous). Analyses of men and women combined also adjusted for sex.

Tests for trend were conducted by modeling the median consumption of coffee in each category as a continuous variable. In addition, the continuous measure of coffee consumption (cups/d) was used to fit a restricted cubic spline model and to obtain a smooth representation of the RR as a function of coffee consumption. We used three knots to divide the continuous coffee consumption into four intervals.

We performed sensitivity analyses by excluding those with a history of cardiac disease or hypertension because they might have changed their coffee consumption before baseline. Furthermore, we examined the effect of excluding coffee abstainers from the reference group or excluding AF cases diagnosed during the first two years of follow-up. We also conducted an analysis using coffee abstainers as the reference group. All statistical analyses were conducted using SAS (version 9.3; SAS Institute, Cary, NC, USA). Two-sided *P* values <0.05 were considered statistically significant.

### Meta-analysis

We conducted a dose–response meta-analysis that included results from our two prospective cohorts (COSM and SMC) as well as findings from previously published prospective studies of coffee consumption and AF. Studies were identified by a computerized search of PubMed and Embase through 22 July 2015, and by reviewing the reference lists of retrieved articles and previous meta-analyses on caffeine intake and AF [11, 12]. We used the search terms ‘coffee’ or ‘hot beverages’ or ‘diet’ combined with ‘atrial fibrillation’ or ‘flutter’ or ‘arrhythmia’. No restrictions were imposed. Studies were eligible for inclusion in the meta-analysis if they: 1) had a prospective design; 2) the exposure was coffee consumption; 3) the outcome was incidence of AF or AF and atrial flutter combined; and 4) RRs with 95 % CIs were reported.

From every study, we extracted the first author’s last name, publication year, country of origin, sex and age of study participants, years of follow-up, method to ascertain AF events, covariates adjusted for in the analysis, and RRs with 95 % CIs (from the most fully adjusted model) for each category of coffee consumption. We also extracted the total number of cases and participants as well as the number of cases and participants in each exposure category.

We combined the study-specific RRs for the highest versus the lowest category of coffee consumption using a random-effects model, which considers both within- and between-study variability. Subgroup analyses by country and sex were conducted. In a sensitivity analysis, we added studies that reported results on caffeine (but not coffee) intake in relation to risk of AF. We performed a random-effects dose–response meta-analysis using the same method as described in previous meta-analyses [4, 5] to compute the RRs per 2 cups/d increment of coffee consumption. Statistical heterogeneity was investigated using the *P* and *I*^*2*^ statistics [18]. Publication bias was assessed with Egger’s test [19]. We used Stata (version 12.0, StataCorp, College Station, TX, USA) for the statistical analyses.

## Results

### COSM and SMC

Compared with those with a low coffee consumption, men and women who daily consumed 5 or more cups of coffee were slightly younger, had lower education, and were more likely to be current smokers but less likely to have a history of cardiac disease, hypertension and diabetes (Table 1). They also consumed less tea. The median daily coffee consumption was 3 cups among both men and women. Participants who were excluded from the analyses because of a diagnosis of AF at baseline consumed less coffee (median 2.1 cups/d).

**Table 1:** Baseline age-standardized characteristics of men in the Cohort of Swedish Men and women in the Swedish Mammography Cohort by categories of coffee consumption^a^

	Coffee consumption, cups/day									
	Men (*n* = 41,881)					Women (*n* = 34,594)				
	<2	2 to <3	3 to <4	4 to <5	≥5	<2	2 to <3	3 to <4	4 to <5	≥5
	*n* = 6,605	*n* = 10,017	*n* = 7,804	*n* = 7,342	*n* = 10,111	*n* = 5,745	*n* = 9,552	*n* = 7,656	*n* = 6,017	*n* = 5,624
Age (years)	61 (9.6)	61 (9.8)	61 (9.8)	60 (9.7)	57 (8.9)	62 (9.4)	62 (9.4)	62 (9.3)	61 (9.2)	59 (9.1)
Postsecondary education (%)	20	18	18	15	12	24	21	19	16	15
Current smokers (%)	18	21	21	26	37	16	19	22	27	37
History of cardiac disease (%)	10	9.7	9.1	8.6	8.9	5.3	4.0	4.3	4.5	4.1
History of hypertension (%)	28	25	24	23	22	23	23	21	20	20
History of diabetes (%)	10	9.4	9.0	9.0	9.6	5.1	4.9	4.4	4.3	4.6
Walking/bicycling ≥20 min/day (%)	61	63	63	64	60	70	71	71	71	68
Family history of MI (%)	15	15	14	15	16	17	17	17	17	18
Body mass index (kg/m^2^)	26 (3.4)	26 (3.2)	26 (3.1)	26 (3.1)	26 (3.3)	25 (4.0)	25 (3.8)	25 (3.7)	25 (3.8)	25 (3.9)
Alcohol intake (drinks/week)^b^	8.0 (7.3)	8.0 (6.8)	7.8 (6.7)	7.8 (6.8)	7.7 (7.1)	4.0 (4.6)	3.8 (4.1)	3.7 (4.0)	3.6 (3.9)	3.6 (4.0)
Tea consumption (cups/day)	1.1 (1.4)	0.7 (1.0)	0.5 (0.9)	0.4 (0.9)	0.3 (0.8)	1.1 (1.3)	0.7 (1.0)	0.5 (0.9)	0.4 (0.8)	0.3 (0.9)

^a^Baseline characteristics are presented as mean (± SD) or %; ^b^among current drinkers. MI, myocardial infarction

During 12 years of follow-up, AF occurred in 4,311 men during 449,744 person-years and in 2,730 women during 382,000 person-years. Coffee consumption was not associated with risk of AF, although there was a non-significant positive association in men and a non-significant inverse association in women (Table 2). When we used splines to examine more extreme levels of coffee consumption, we observed no significant association in either men or women or in men and women combined (Additional file 1: Web Figure S1). The results remained essentially the same after removing those with a history of cardiac disease and/or a history of hypertension at baseline, or removing coffee abstainers (data not shown). In women, the RR of AF comparing the highest with the lowest category of coffee consumption was slightly attenuated after exclusion of AF cases diagnosed during the first two years of follow-up (RR = 0.91; 95 % CI 0.78–1.06). When we used coffee abstainers as the reference group (only 1.2 % of the study population; *n* = 72 cases), the multivariable RR of AF was 0.92 (95 % CI 0.72–1.17).

**Table 2:** Association between coffee consumption and incidence of atrial fibrillation among men in the Cohort of Swedish Men and women in the Swedish Mammography Cohort, 1998−2009

	Coffee consumption, cups/day					
	<2 (1)^a^	2 to <3 (2)	3 to <4 (3)	4 to <5 (4)	≥5 (6)	*P* trend
Men (*n* = 41,881)						
Number of cases	742	1,155	813	715	886	
Person-years	69,946	106,314	83,602	78,990	110,892	
Age-adjusted RR (95 % CI)	1.00	0.97 (0.88–1.06)	0.93 (0.84–1.03)	0.90 (0.81–1.00)	1.02 (0.92–1.12)	0.74
Multivariable RR (95 % CI)^b^	1.00	0.99 (0.90–1.09)	0.99 (0.89–1.09)	0.97 (0.87–1.07)	1.08 (0.98–1.20)	0.10
Women (*n* = 34,594)						
Number of cases	505	804	617	473	331	
Person-years	62,699	104,866	84,552	66,627	63,256	
Age-adjusted RR (95 % CI)	1.00	0.94 (0.84–1.05)	0.95 (0.85–1.07)	0.96 (0.85–1.09)	0.89 (0.77–1.02)	0.17
Multivariable RR (95 % CI)^b^	1.00	0.95 (0.85–1.07)	0.98 (0.87–1.10)	0.98 (0.86–1.12)	0.88 (0.76–1.02)	0.17
Men and women combined						
Multivariable RR (95 % CI)^b^	1.00	0.98 (0.91–1.05)	0.99 (0.91–1.07)	0.97 (0.90–1.05)	1.01 (0.93–1.10)	0.64

^a^Median consumption in parenthesis; ^b^adjusted for age, education, smoking, histories of cardiac disease, hypertension, diabetes, body mass index, walking/bicycling, family history of myocardial infarction, and consumption of alcohol and tea. Results for men and women combined are also adjusted for sex. CI, confidence interval; RR, relative risk

### Meta-analysis

Six prospective studies (including COSM and SMC) [20–23], including a total of 10,406 AF cases (68 % of the cases came from COSM and SMC) diagnosed among 248,910 individuals, were eligible for inclusion in the meta-analysis (Additional file 1: Web Figure S2; flow chart). Four studies were conducted in Sweden and two in the US (Table 3). Coffee consumption was assessed via a self-administered questionnaire in all studies.

**Table 3:** Characteristics of prospective studies included in the meta-analysis of coffee consumption and atrial fibrillation

First author, year; study name	Country	Population	Follow-up (years)	Outcome assessment	Number of cases^a^	Category of coffee^b^	Adjusted RR (95 % CI)	Adjustment
Wilhelmsen, 2001 [20]; Multifactor Primary Prevention Study	Sweden	7,374 men, 47–55 years of age	27	ECG, hospital records and register (ICD-9 code 427D)	754	0	1.00	Age
						1–4	1.24 (1.00–1.54)	
						≥5	1.09 (0.87–1.38)	
Mukamal, 2009 [21]; Stockholm Heart Epidemiology Program	Sweden	1,369 men and women, 45–70 years of age and who had survived a MI	9.9	Register (ICD-9 code 427D and ICD-10 code I48)	163	0 to <1	1.00	Age, sex, education, smoking, diabetes, obesity, physical inactivity, and intake of alcohol, tea and boiled coffee
						1 to <3	0.71 (0.42–1.20)	
						3 to <5	0.61 (0.35–1.04)	
						5 to <7	0.61 (0.34–1.10)	
						≥7	0.67 (0.33–1.34)	
Conen, 2010 [22]; Women’s Health Study	USA	33,638 women ≥45 years of age and free from AF and CVD	14.4	Self-reported and confirmed by medical record review	936	0	1.00	Age, race/ethnicity, treatment group, systolic blood pressure, hypertension, hypercholesterolemia, smoking, diabetes, BMI, exercise, parental history of myocardial infarction, and intake of alcohol and fish
						<1	1.03 (0.87–1.21)	
						1	0.93 (0.71–1.21)	
						2–3	1.36 (1.12–1.65)	
						≥4	1.03 (0.79–1.35)	
Klatsky, 2011 [23]; California Comprehensive Health Care Plan	USA	130,054 men and women; age NA	17.6	Register (ICD-9 code 427.31)	1,512	0	1.00	Age, sex, ethnicity, BMI, education, cigarette smoking, a cardiorespiratory composite covariate, and alcohol intake
						<1	0.82 (0.67–1.00)	
						1–3	0.88 (0.76–1.01)	
						≥4	0.81 (0.69–0.96)	
Larsson, 2015; Cohort of Swedish Men (current study)	Sweden	41,881 men, 45–79 years of age and free from AF	12	Register (ICD-10 code I48)	4,311	<2	1.00	Age, education, smoking, histories of cardiac disease, hypertension and diabetes, BMI, walking/bicycling, family history of MI, and intake of alcohol and tea
						2 to <3	0.99 (0.90–1.09)	
						3 to <4	0.99 (0.89–1.09)	
						4 to <5	0.97 (0.87–1.07)	
						≥5	1.08 (0.98–1.20)	
Larsson, 2015; Swedish Mammography Cohort (current study)	Sweden	34,594 women, 49–83 years of age and free from AF	12	Register (ICD-10 code I48)	2,730	<2	1.00	Age, education, smoking, histories of cardiac disease, hypertension and diabetes, BMI, walking/bicycling, family history of MI, and intake of alcohol and tea
						2 to <3	0.95 (0.85–1.07)	
						3 to <4	0.98 (0.87–1.10)	
						4 to <5	0.98 (0.86–1.12)	
						≥5	0.88 (0.76–1.02)	

^a^Number of cases included in the analysis of coffee consumption and atrial fibrillation; ^b^coffee consumption in cups/day. AF, atrial fibrillation; BMI, body mass index; CI, confidence interval; CVD, cardiovascular disease; ECG, electrocardiogram; ICD, International Classification of Disease; MI, myocardial infarction; NA, not available; RR, relative risk

**Fig. 1:**
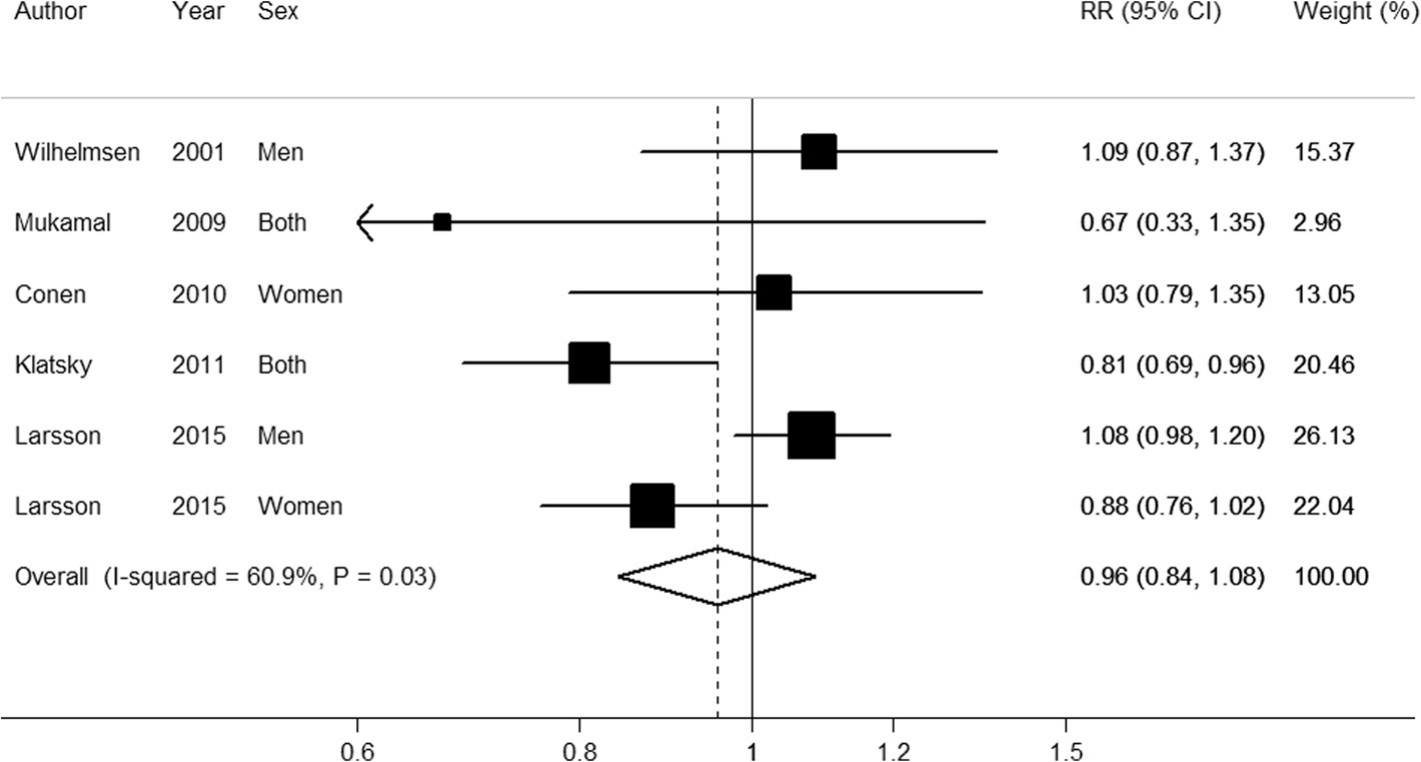
Forest plot of the relative risks (RR) of atrial fibrillation for the highest versus lowest category of coffee consumption (cups/day). Squares indicate study-specific RR estimates (size of the square reflects the study-specific statistical weight); *horizontal lines* indicate the 95 % CI; *diamond* indicates the overall RR with its 95 % CI. Study-specific RRs were combined using a random-effects model

Of the six studies, one study [23] showed a statistically significant reduced risk of AF when comparing the highest with the lowest category of coffee consumption, but there was no dose–response relationship; the other five studies observed no significant association (Fig. 1). When results from all studies were combined, the RR of AF comparing the highest with the lowest category of coffee consumption was 0.96 (95 % CI 0.84–1.08), with heterogeneity among studies (*I*^2^ = 60.9 %). Excluding the study that did not adjust for smoking [20] did not alter the results materially (RR = 0.93; 95 % CI 0.80–1.08; *I*^2^ = 66.2 %). In stratified analyses, the RRs were 0.89 (95 % CI 0.71–1.12; *I*^2^ = 55.3 %) for studies conducted in the US (two studies), 0.99 (95 % CI 0.86–1.15; *I*^2^ = 55.7 %) for studies conducted in Sweden (four studies), 1.08 (95 % CI 0.99–1.19; *I*^2^ = 0 %) in men (two studies) and 0.91 (95 % CI 0.80–1.04; *I*^2^ = 1.8 %) in women (two studies). In a sensitivity analysis, we included two studies that assessed caffeine intake [24, 25]. In this analysis, which included six studies of coffee consumption and two studies of caffeine intake in relation to AF risk, the overall RR for the highest versus lowest category of coffee or caffeine intake was 0.95 (95 % CI, 0.86–1.06; *I*^2^ = 46.3 %).

There was no evidence of a nonlinear relationship between coffee consumption and AF risk (*P* nonlinearity = 0.55). The RR per 2 cups/d increment of coffee consumption was 0.99 (95 % CI 0.94–1.03; *I*^2^ = 65.7 %) (Additional file 1: Web Figure S3). We found no indication of publication bias (*P* ≥0.43).

## Discussion

In the COSM and the SMC, coffee consumption was not associated with incidence of AF. Likewise, results from a complementary meta-analysis showed no overall association between coffee consumption and AF risk, but there was heterogeneity among studies. All studies were conducted in either Sweden or the US, thus reducing the generalizability of the results.

In sex-specific analyses, coffee consumption was associated with a non-significant positive association in men, but with a non-significant inverse association in women. Whether men may be more sensitive to a high coffee or caffeine intake warrants further study.

Although available evidence does not indicate that coffee consumption increases the risk of developing AF, coffee (or caffeine) may trigger arrhythmia. In a study of 100 patients with idiopathic paroxysmal AF, 25 patients indicated coffee consumption as a triggering factor for arrhythmia [26]. In the COSM and the SMC, participants who had AF at baseline consumed, on average, less coffee than those without AF, suggesting that some individuals with AF may have quit drinking coffee or lowered their consumption because of an arrhythmic-triggering effect of coffee.

Strengths of the COSM and the SMC are the large sample sizes and the large number of incident AF cases. Moreover, the accuracy of the AF diagnosis in the Swedish Hospital Discharge Register is high [17]. However, the AF cases in our cohorts are mainly symptomatic cases. We cannot exclude the possibility that bias might have been introduced if individuals with first episodes of less serious AF (undiagnosed cases) might have reduced their consumption of coffee. Because of the observational nature of our studies, we cannot rule out confounding as a potential explanation for the lack of association between coffee consumption and AF risk, although we adjusted for major AF risk factors. In addition, because coffee consumption was assessed with a self-administered questionnaire and only at baseline, some measurement error in the assessment of coffee consumption was inevitable, and could explain our null results. Another limitation is that we did not have information on type of coffee (for example, decaffeinated) and preparation method (for example, filtered or boiled), and we had limited statistical power in our analysis using coffee abstainers as the reference. The meta-analysis inherits the limitations of the included studies. The limitations in the other studies are about the same as those discussed for the COSM and the SMC. Publication bias could be of concern in any meta-analysis of published data. We observed no evidence of such bias in the present meta-analysis.

Two recent meta-analyses of the association between caffeine intake and AF risk showed no overall association [11, 12]. In one of those meta-analyses, which included six prospective studies, there was a statistically significant inverse association between caffeine intake and AF in a subgroup analysis of four studies that adjusted for smoking [11]. In the other meta-analysis, including six prospective studies and one case–control study, low caffeine intake but not moderate and high intakes was associated with a reduced risk of AF [12].

## Conclusions

Available evidence from prospective studies indicates that coffee consumption is not associated with risk of AF. However, as the number of studies of coffee consumption and AF risk is quite limited, more large prospective studies investigating this relationship are needed.

## Additional file

Additional file 1: Web Figure S1. Multivariable relative risks of atrial fibrillation by coffee consumption (in men in the COSM and in women in the SMC) modeled with restricted cubic splines. The relative risks are plotted on the log scale. Dashed lines represent the 95 % confidence intervals for the spline model. Consumption of 2 cups/d served as the reference group. **Web Figure S2.** Flow chart of study selection. **Web Figure S3.** Relative risks of atrial fibrillation by coffee consumption in a dose-response meta-analysis of six prospective studies. The relative risks are plotted on the log scale. Dashed lines represent the 95 % confidence intervals. Consumption of 0 cups/d served as the reference group. (DOCX 65 kb)

## Abbreviations

AF: Atrial fibrillation
CI: Confidence interval
COSM: Cohort of Swedish Men
ICD: International Classification of Disease
RR: Relative risk
SMC: Swedish Mammography Cohort
