# Supplementary material for: Coffee consumption is not associated with increased risk of atrial fibrillation: results from two prospective cohorts and a meta-analysis
Source: BMC Med. 2015 Sep 23;13:207. doi: 10.1186/s12916-015-0447-8 (PMC4579587; doi:10.1186/s12916-015-0447-8)
Supplement: Additional file 1: Web Figure S1. — Multivariable relative risks of atrial fibrillation by coffee consumption (in men in the COSM and in women in the SMC) modeled with restricted cubic splines. The relative risks are plotted on the log scale. Dashed lines represent the 95 % confidence intervals for the spline model. Consumption of 2 cups/d served as the reference group. Web Figure S2. Flow chart of study selection. Web Figure S3. Relative risks of atrial fibrillation by coffee consumption in a dose-response meta-analysis of six prospective studies. The relative risks are plotted on the log scale. Dashed lines represent the 95 % confidence intervals. Consumption of 0 cups/d served as the reference group. (DOCX 65 kb) [file 12916_2015_447_MOESM1_ESM.docx]

**Web Figure S1 Multivariable relative risks of atrial fibrillation by coffee consumption (in men in the COSM and in women in the SMC) modeled with restricted cubic splines.** The relative risks are plotted on the log scale. Dashed lines represent the 95% confidence intervals for the spline model. Consumption of 2 cups/d served as the reference group.

**Web Figure S2 Flow chart of study selection**


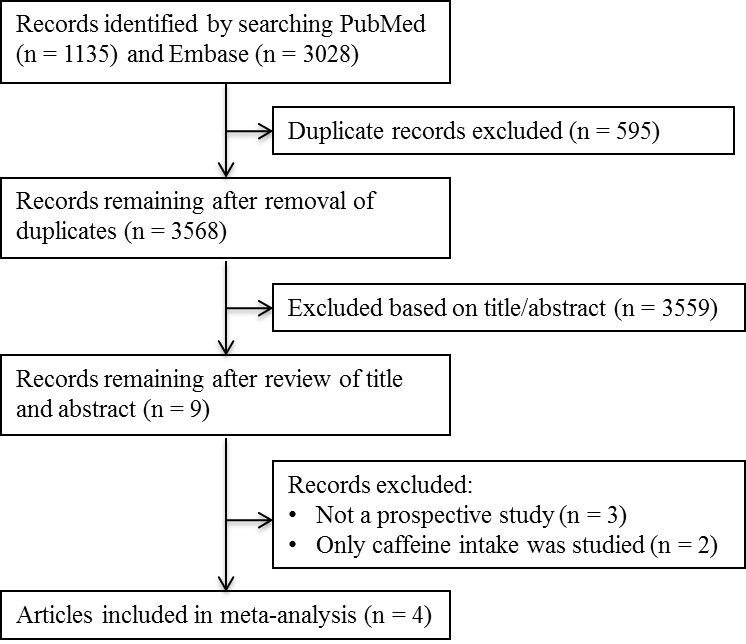


**Web Figure S3 Relative risks of atrial fibrillation by coffee consumption in a dose-response meta-analysis of six prospective studies.** The relative risks are plotted on the log scale. Dashed lines represent the 95% confidence intervals. Consumption of 0 cups/d served as the reference group.
